# No Evidence for Memory Decontextualization across One Night of Sleep

**DOI:** 10.3389/fnhum.2016.00007

**Published:** 2016-01-26

**Authors:** Katarzyna Jurewicz, Maren Jasmin Cordi, Tobias Staudigl, Björn Rasch

**Affiliations:** ^1^Department of Neurophysiology, Nencki Institute of Experimental BiologyWarsaw, Poland; ^2^Division of Biopsychology, Institute of Psychology, University of ZurichZurich, Switzerland; ^3^Zurich Center for Interdisciplinary Sleep Research (ZiS), University of ZurichZurich, Switzerland; ^4^Department of Psychology, University of KonstanzKonstanz, Germany; ^5^Donders Institute for Brain, Cognition and Behaviour, Radboud University NijmegenNijmegen, Netherlands; ^6^Department of Psychology, Division of Cognitive Biopsychology and Methods, University of FribourgFribourg, Switzerland

**Keywords:** sleep, memory, context, decontextualization, consolidation

## Abstract

Sleep after learning strengthens memory consolidation. According to the active system consolidation hypothesis, sleep supports the integration of newly acquired memories into cortical knowledge networks, presumably accompanied by a process of decontextualization of the memory trace (i.e., a gradual loss of memory for the learning context). However, the availability of contextual information generally facilitates memory recall and studies on the interaction of sleep and context on memory retrieval have revealed inconsistent results. Here, we do not find any evidence for a role of sleep in the decontextualization of newly learned declarative memories. In two separate studies, 104 healthy young adults incidentally learned words associated with a context. After a 12 h retention interval filled with either sleep or wakefulness, recall (Experiment 1) or recognition (Experiment 2) was tested with the same or different context. Overall, memory retrieval was significantly improved when the learning context was reinstated, as compared to a different context. However, this context effect of memory was not modulated by sleep vs. wakefulness. These findings argue against a decontextualization of memories, at least across a single night of sleep.

## Introduction

Sleep benefits episodic memories (Rasch and Born, 2013). Numerous studies consistently report that recall of episodic memories is better after sleep as compared to a retention period filled with wakefulness (e.g., Plihal and Born, 1997; Ellenbogen et al., 2009). According to the active system consolidation hypothesis (Diekelmann and Born, 2010) spontaneous reactivations of recently acquired memories during sleep mediate their reorganization and integration into long-term memory networks. This process is assumed to change the extent to which they rely upon hippocampus and parahippocampal structures towards a greater dependency on the neocortex (Frankland and Bontempi, 2005). It is hypothesized that through a selective reactivation of some central aspects of experiences (a memory’s gist) at the expense of weakly associated additional information (such as e.g., the learning context), overlapping aspects of different memories are reactivated over and over again during sleep and establish a cognitive schema independent from source memory (Lewis and Durrant, 2011). Thereby, a qualitative transformation from perceptually rich and detailed episodic memories towards more abstract semantic knowledge might be supported by sleep, a process, which could be called “semantization” or “decontextualization”. Based on these assumptions, sleep is predicted to foster “decontextualization”, thereby actively supporting the gradual decrease of association strength between the gist and the contextual information of episodic memories.

One possibility to experimentally examine the association strength between memory content and context is provided by the well-known context effect of memory (Smith and Vela, 2001; Aslan et al., 2010). In brief, contextual cues improve memory recall when they overlap with cues, which were available during prior memory encoding. The context effect of memory emerges as during encoding, the central aspects of newly remembered experience are stored together with the contextual setting present during acquisition. During later retrieval, re-exposure to the same contextual information facilitates recall as opposed to a different retrieval context (e.g., Godden and Baddeley, 1975, 1980; Aslan et al., 2010). Different studies have already reported such an effect for a wide range of contextual cues such as experimental room (e.g., Smith, 1979), odor (e.g., Schab, 1990), background music (e.g., Smith, 1985), the combination of the previous three (e.g., Parker et al., 2007), the visual background of stimuli (e.g., Isarida and Isarin, 2007) or mood (e.g., Kenealy, 1997). As the hippocampus is critically involved in binding contextual features to episodic memories (Davachi, 2006), this brain region is assumed to mainly support the context effect on memory (Staudigl and Hanslmayr, 2013). It is important to note that the stimuli predominantly used in the studies of context effect, i.e., words, are already integrated into a semantic association network. Thus, their occurrence in a specific, task-irrelevant context will only weakly create new associations between the word and the learning context. According to Lewis and Durrant (2011), such weak associations between a learning context and a familiar word are weakening or even lost during sleep.

Interestingly, in spite of the theoretical assumption that episodic memories are generally “decontextualized” over time including sleep, the size of the context effect on memory considerably increases with longer test intervals (for a meta-analysis, see Smith and Vela, 2001). Particularly after intervals between learning and testing of 1 day to 1 week, high context reinstatement effects were reported. This result indirectly suggests that longer retention intervals including sleep periods increase rather than decrease the influence of contextual cues on memory retrieval. In line with this notion, two previous studies that more specifically examined the effect of sleep on item-related and contextual information of visual scenes even reported improved memory for contextual information after a nap (van der Helm et al., 2011) as well as after a whole night of sleep (Lewis et al., 2011). However, participants were explicitly instructed to associate items with their backgrounds, which might have generated associative memory traces rather than incidental encoding of the learning contexts. Additionally, the assigned relevance of a memory as well as its initial trace strength can determine which memories are consolidated (Fischer and Born, 2009; Tucker et al., 2011; van Dongen et al., 2012). Thus, possibly relevant or deeply encoded context memory does not or only hardly suffer from decontextualization. In a very recent study, Cox et al. (2014) tested the effects of incidentally encoded context on word stem completion after previous word-photo pair learning. The first recall of half of the items took place immediately, the second, including the other half of the learned items after 12 h filled with sleep or wakefulness or after 24 h. Thereby, the words stems were either presented with the same background as during learning or the word-photo pairs were shuffled and re-paired. Interestingly, results demonstrated a time- but not sleep-dependent reduction of context effects. Thus, the congruent items were more rapidly forgotten between sessions, which was however independent from sleep or wakefulness during the retention interval. This result not only contradicts the time-dependent increase in context effect size reported in the meta-analysis by Smith and Vela (2001), but also the decontextualization assumption derived from the active system consolidation hypothesis.

There is however one study which reported data in line with the prediction of a role of sleep in memory decontextualization. In this study by Cairney et al. (2011), participants learned two word lists in two different rooms (i.e., room 1 and 2). Immediate recall of both lists took place in one of these rooms (e.g., room 1). After 12 h filled with sleep or wakefulness, they again recalled both lists in this last room (e.g., room 1). Comparing immediate and delayed recall showed that if the retention interval was filled with wakefulness, participants recalled more words learned in the same (e.g., room 1) as compared to those learned in a different context (room 2). In contrast, no beneficial effect of the context on memory retrieval was observed in the sleep group. This finding was interpreted as a “decontextualization” of memories through sleep, indicating that sleep reduces the degree to which context influences memory performance. However, note that both, immediate recall and delayed recall, were tested in an identical context (e.g., room 1), thus no context change occurred across the retention interval. Thus, an alternative explanation for the result pattern is that during immediate recall before the retention interval (i.e., sleep vs. wakefulness), a new association was created between words originally learned in a different context (e.g., room 2) and the current immediate recall context (e.g., room 1). This new association might have been particularly strengthened during sleep, because sleep preferentially consolidates memories acquired shortly before sleep (Gais et al., 2006). Thus, after a retention interval filled with sleep, both word categories are similarly well associated with the immediate recall context (e.g., room 1), and memory retrieval does not depend on the initial learning context anymore. In contrast, across wakefulness, this new association was not particularly strengthened, resulting in a better recall of words initially learned in the same as compared to a different context. Thus, introduction of an immediate recall session before the retention interval filled with either sleep or wakefulness might have confounded the results.

In the view of these inconsistent and inconclusive findings, here we aimed at testing the interaction between context and sleep in a well-established context reinstatement paradigm in two separate experiments. One hundred four healthy young participants viewed short movies in the background of displayed words. The memory for words was later tested and movies served as contextual cues. After 12 h of night time sleep or day time wakefulness, an unexpected recall test (Experiment 1) or recognition test (Experiment 2) was performed using matching and/or non-matching word/video pairs. Generally, sleep improved memory for words in the recall test, independent of the context condition. Additionally, in both experiments, memory performance was significantly improved in the matching context condition as compared to non-matching word/video pairs. However, sleep vs. wakefulness after encoding did not alter the beneficial effect of context reinstatement on memory performance, indicating that sleep did not decontextualize memories.

## Materials and Methods

### Participants

In Experiment 1, 57 healthy adults (mean age 22.51 ± 2.92 (SD), range 19–42 years) were recruited at the University of Warsaw and from academic websites. In Experiment 2, 47 healthy adults (mean age 24.11 ± 3.56, range 19–32 years) were recruited from the University of Zurich and its websites. Participants were randomly assigned to one of two groups (“wake” and “sleep”, see “General Procedure” Section)—in Experiment 1, 28 (9 male) and 29 (12 male) subjects were enrolled, respectively. In Experiment 2, 24 (8 male) subjects took part in the wake group, 23 (8 male) in the sleep group. All subjects gave their written informed consent. The local ethics committee of Zurich approved the experiments. Participants were medication-free, had no history of neurological or psychiatric disorders, had normal sleep (a Pittsburgh Sleep Quality Index (PSQI) score of ≤7 was chosen as a cut-off value) and were not subjected to any conceivable shifts in their habitual sleep-wake rhythm during 4 weeks prior to the experiment. Participants were told that the study goal was to assess the influence of circadian factors upon cognitive processing. They were paid 40 PLN (~13 USD, Experiment 1) or 30 CHF (~33 USD, Experiment 2), respectively for participating in two experimental sessions. They were asked to abstain from caffeine and alcohol on experimental days and to avoid any naps between the sessions. One subject was considered an outlier with respect to memory performance (>3 standard deviations (SD) below the group mean) and was excluded from Experiment 2.

### General Procedure

**Figure 1:**
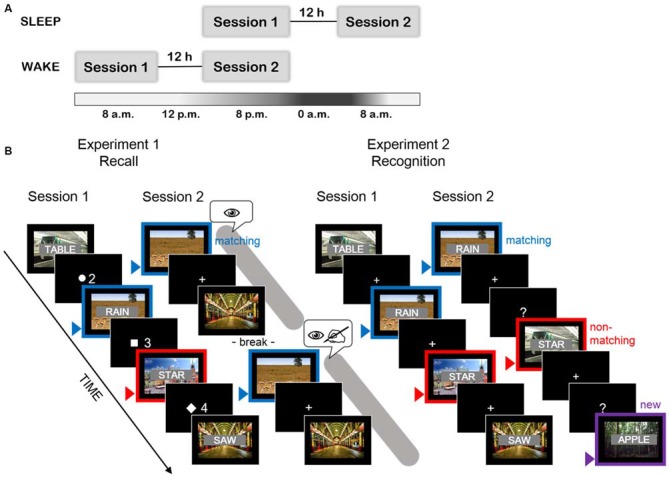
**(A)** Session flow for both experiments. Memory for words was tested in a between-subjects design, comparing the two groups: sleep group (upper part of the scheme), having the encoding in the evening and the test in the morning and wake group (lower part of the scheme), having the reversed order of sessions. **(B)** Schematic representation of the task design in both experiments. Blue arrows and frames indicate word/video pairs, which were subjected to contextual reinstatement in the second session (by exposition of videos in Experiment 1 or identical word/video pairs in Experiment 2). Red arrows and frames indicate a trial, in which the videos were not reinstated in the second session (Experiment 1: all odd or even half of the trials) or correspond to trials with non-matching word/video pairing in the second session (Experiment 2). Violet arrow indicates a new word/video pair, not present in the first session (to control for false positive recognitions, only in Experiment 2). The symbols used in recall session of Experiment 1 (eye, writing hand) indicate the instructions provided before the first and the second presentation of the videos. First, participants were asked only to watch the videos. Later, they were asked to recall all the words from the Session 1 and to write them down on the answer-sheet while having the videos played once again. The time dedicated to completion of both instructions in Experiment 1 is marked by the gray area. The time for completion of the recognition task in Experiment 2 is represented by the screens with question marks.

The encoding session took either part in the evening and memory was tested in the morning, after a night at home (“sleep group”) or the encoding session was in the morning and the memory test was conducted in the evening after a day of normal daily activity (“wake group”) (see Figure 1A). The morning sessions started between 7 and 9 a.m., the evening sessions between 7 and 9 p.m. In the first session, subjects filled out questionnaires concerning sleep behavior, alcohol, and tobacco consumption and the standardized mood questionnaire. Thereafter, they performed the declarative memory task before they were confronted with a working memory task (mathematical task in Experiment 1 or N-back task in Experiment 2). In the second session, participants answered the same mood questionnaire. Afterwards, an unannounced (surprise) memory test was provided, followed by the working memory task. In the end they answered questions about the experiment and the debriefing was done.

### Questionnaires

To assess subjective sleep quality, German and Polish versions of the PSQI (Buysse et al., 1989) were used. The index of the PSQI ranges from 0 to 21, with a higher score indicating worse sleep. To assess participants’ psychophysical state (i.e., perceived mood, alertness and tension) at the time of task completion, the Polish adaptation of the UWIST Mood Adjective Checklist (UMACL; Matthews et al., 1990; Gorynska, 2005) was applied in Experiment 1 and the Mehrdimensionaler Befindlichkeitsfragebogen (MBDF) Kurzform A (Buysse et al., 1989) was used in Experiment 2. Both questionnaires assessed good/bad mood, alertness/tiredness and calmness/agitation on a Likert scale (definitely not—extremely). To exclude possible circadian confounds, the cognitive capacity at time of task completion was also evaluated by a working memory task: a mathematical task (Experiment 1), consisting of simple additions in a limited time or the N-back task (Experiment 2) in which participants must indicate whether the current digit equals the one from n steps earlier in a sequence of digits.

### Experiment 1: Recall

#### Stimuli

Using videos as local context to words has been shown to be a sensitive method to measure the influence of context on memory (Smith and Manzano, 2010). Here, we adapted the paradigm published by Smith and Manzano (2010) to examine effects of sleep vs. wakefulness on context effect in recall. Thus, 56 videos were chosen at random from the larger set of videos we used in Experiment 2, a set which has previously been shown to reliably induce context-dependent memory effects (Staudigl and Hanslmayr, 2013). It contained 365 movie and TV program sequences of 3040 ms, provided by the Landesfilmdienst Baden-Württemberg, Germany. Their content was diversified, presenting e.g., landscapes, animals in natural environment, working machines, or human activities. All pieces of videos were soundless, colored, contained some moving features and did not show any obviously emotional or highly distinctive pictures such as high-contrast human faces or highly threatening events. A list of words was constructed similarly to Cairney et al. (2011). It contained 56 Polish nouns, 4 per each of 14 word categories (fishes, mushrooms, kitchen utensils, tissues, occupations, building materials, dances, toys, green objects, liquids, birds, insects, flowers, carpenter tools). The words had a low frequency level from 0.1 to 2.9 per million words according to National Corpus of Polish (Pęzik, 2012) and a length of 3–9 letters. Low frequency level was chosen to prevent from guessing based on category. Words were divided into two lists (A and B), each containing two words from each category, carefully matched for length (*t*_(1,54)_ = 0.44, *p* = 0.73, mean number of letters 6.14 and 6.29). Each word was written in white characters on black background and superimposed centrally on a video (in the following referred to as word/video pair, see Figure 1B, left side). All videos were 360 × 288 pix in size and the words were approximately 1/5 of height of the videos. The initial pairing of words and videos was assigned at random, without any purposeful, obvious relations between word meaning and the content of the video. During later recall testing, half of the videos (corresponding to words from list A or B) were presented again. The words previously studied together with these videos (e.g., list A) were therefore recalled in a reinstated or “matching” context (see Figure 1B, left side). For the other half of the words (e.g., list B) for which the videos were not played again during cued recall testing, the learning context was not reinstated (therefore referred to as “non-matching”).

#### Declarative Memory Task Used for Recall Testing

The tasks were implemented using Psychopy 1.75.01 and displayed on the projection screen. The 56 word/video pairs were presented centrally on the black computer screen and scaled to the projection screen. Participants were asked to rate the pleasantness of the words on a scale from 1 to 5 to achieve strong initial processing. Participants were not instructed to memorize the items, but were told that the study aims at investigating the influence of circadian factors on cognitive processing. All answers were collected in paper-pencil form. To ensure attention and enable the rating only after the presentation, subjects were given an answer sheet with a table of ordinal numbers in rows and three symbols (square, triangle or circle) in columns indicating where to put the answer. The ordinal number according to the stimuli was presented for 1 s before the stimulus, while the referring symbol appeared for 3 s afterwards. Thus, the answer could only be given after having paid attention to the whole presentation. As the correctness of the answers could not be derived from the task, correct answers produced without word processing cannot be excluded. Therefore, simultaneously to visual presentation, each word was presented acoustically. Word/video pairs were presented in a pseudo-random order, which remained the same across subjects. Words from the same category never occurred in direct succession and words from A and B lists were presented in even and odd word/video pairs respectively. In the test phase, participants were confronted only with 24 videos from the first session, played two times in the same order. Either the even or the odd half of all videos (counterbalanced within each group) was taken, related to half of the 48 words remaining after the exclusion of the first and last four words of two categories (to avoid primacy and recency effects). Thus, the maximum score of remembered items was 48. The videos were separated by the presentation of a fixation cross (4.06 s). The first time these 24 videos were played, subjects were instructed to only watch them carefully. This warranted undisturbed re-exposure to the stimuli, refreshing memory traces of the context for half of the words. Afterwards, participants were asked to recall all the words seen in the initial session by writing them down. While participants had time to write down all the words they could remember from the first session, the 24 videos were played again a second time. Subjects were asked to preferably (but not necessarily) write down the words while the fixation cross was present on the screen between each of the videos. To avoid floor effects, participants were provided with an answer sheet with word categories for the seen and unseen words based on Cairney et al. (2011).

### Experiment 2: Recognition

#### Stimuli

To compensate for the ease of recognizing instead of recalling items, all 365 words and videos were used in both sessions of Experiment 2 and encoding was shallow. The list of words consisted of German nouns, as used in Wimber et al. (2010). Concrete and abstract nouns of 3–8 letters were included, with initial letters covering almost all letters of the alphabet. For recognition, contextual manipulation was induced by mere exposure of the same (matching), shuffled (non-matching) and completely new word/video pairs (new).

#### Declarative Memory Task Used for Recognition Testing

The task was implemented using Presentation 14.9 and displayed on 19″ monitor. For each participant, the pairing of words and videos, the order of word/video pairs and the assignment of each pair to one of the three conditions (matching/non-matching/new) were randomized, with the use of MATLAB. During encoding, participants saw 240 word/video pairs, each displayed for 3040 ms and interrupted by a fixation cross presented for 150–650 ms. The task was to indicate whether the first and last letter of the word are in correct alphabetical order (alphabetical order) or not (non-alphabetical order). There were 120 words of each type. Participants responded by pressing either the left or right “Alt” button on the keyboard, with counterbalanced assignment in each group. As before, subjects were not instructed to memorize the stimuli, but only informed that the study aims at investigating the influence of circadian factors on cognitive processing. During the test phase, a recognition task was used to assess memory for the words. Again, word/video pairs were displayed for 3040 ms and for each word, participants were asked whether they had seen it in the previous session or not (see Figure 1B, right side). Therefore, a screen presenting the words “Old” and “New” on the sides was shown. For half of the participants “Old” was shown at the left and “New” on the right, for the other half the direction was reversed to control for left vs. right hand button presses. The screen was displayed until participants gave their response by typing the corresponding numbers on the computer keyboard. Additionally, they indicated for each answer how confident they were about their reply on three consecutive buttons representing three confidence levels (high, medium, and low). After the response, the fixation cross appeared for 150–650 ms before the next word/video pair was presented. After five training trials, 360 word/video pairs were presented, out of which 120 were exactly the same as in the encoding session (matching context), 120 contained the old words and videos but differently paired than in the encoding session (non-matching context) and 120 word/video pairs with new words and videos (new; see Figure 1B, right side). The maximum score at retrieval was thus 120 words per condition.

### Statistical Analysis

A mixed analysis of variance (ANOVA) with the between-subject factors “group” (sleep vs. wake), “gender” (male vs. female) and the within-subject factor “context” (matching vs. non-matching) was used to measure effects on the dependent variable “memory performance”. *Post hoc* pair-wise comparisons were conducted in the case of significant interaction effects and to examine circadian measures. A *p*-value of *p* < 0.05 was considered significant. We included the factor “gender” to control for gender-related differences in verbal and visual recognition abilities (cf. Loftus et al., 1987; McGivern et al., 1997, 1998; Andreano and Cahill, 2009). In recall (Experiment 1), the number of recalled words was taken as dependent measure. For recognition (Experiment 2), the dependent measure (accuracy of recognition) was defined as a sensitivity index *d*′, calculated from *z* transformation of hit (*H*) and false-alarm (F) rates, *d*′ = *z(H)* − *z(F)* (standard deviation units). Standard signal detection theory assumes that participants have a fixed sensitivity, but may change in their readiness to answer “old” or “new” in dubious cases. Therefore an additional, response-bias statistics (criterion location *c*) was calculated, which follows from the equation *c* = −1/2 [*z(H)* + *z(F)*] (Macmillan and Creelman, 2005).

## Results

### Experiment 1 (Recall)

**Figure 2:**
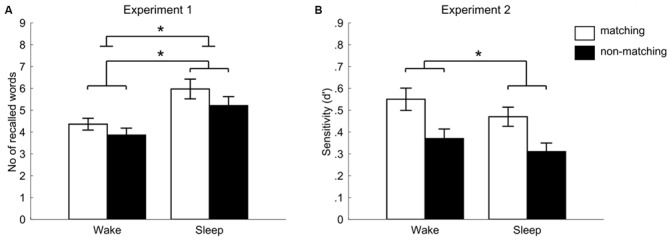
**Memory performance in the two context conditions for sleep and wake groups for Experiment 1 (recall) and Experiment 2 (recognition).** White bars indicate the matching context condition, black bars the non-matching context condition. **(A)** Shows the number of recalled words (Experiment 1) separately for wake and sleep groups (total number of words: 24 in each condition). Significance bars indicate the main effect for group and for context. Importantly, the group × context interaction was non-significant (*F*_(1,53)_ = 0.28, *p* = 0.60, *η*^2^ = 0.005). **(B)** Shows the sensitivity index *d*′ achieved in the recognition task (Experiment 2) separately for wake and sleep groups. The main effect of the “sleep vs. wakefulness” group was non-significant here, while the main effect of context was significant. Also here, we did not find an interaction effect between “sleep vs. wakefulness” and context on recognition performance (*F*_(1,42)_ = 0.09, *p* = 0.77, *η*^2^ = 0.002). Error bars indicate standard error of the mean (SEM). *p < 0.05.

Participants who were allowed to sleep after learning (sleep group) recalled significantly more words [11.17 ± 1.13 words (mean ± SEM)] after the 12 h retention interval as compared to the participants who stayed awake after learning (wake group, 8.21 ± 0.77 words, main effect of “group” *F*_(1,53)_ = 5.02, *p* = 0.029, *η*^2^ = 0.087). Additionally, we observed a significant main effect of context on word recall (*F*_(1,53)_ = 4.29, *p* = 0.043, *η*^2^ = 0.075). Words presented in a matching context were remembered better than those, which were paired to videos not revived at retrieval (5.18 ± 0.39 vs. 4.54 ± 0.38, respectively). However, the interaction between group and context condition was not significant (*F*_(1,53)_ = 0.28, *p* = 0.60, *η*^2^ = 0.005, see Figure 2A): participants in the sleep group remembered more words than participants in the wake group in the matching context (5.97 ± 0.63 vs. 4.36 ± 0.39, sleep vs. wake group, respectively) as well as in the non-matching context (5.21 ± 0.58 vs. 3.86 ± 0.46). There were no differences in memory performance between males and females, and no interaction of gender with other factors (all *p* ≥ 0.24). A postexperimental questionnaire indicated that only 4 out of 57 subjects had expected a memory task in the second session.

### Experiment 2 (Recognition)

In the second experiment, we aimed at replicating our results of the first experiment by testing memory with a recognition paradigm. This was done as context-dependent memory effects do not only occur in recognition (e.g., see Staudigl and Hanslmayr, 2013), but recognition might even be more sensitive to subtle interactions between sleep and context than recall. The performance level in the alphabetical task at encoding was on average 77% correct and there was no difference between wake and sleep group in this task (77.54 ± 2.84 vs. 76.72 ± 2.76, *t*_(1,44)_ = 0.21, *p* = 0.84). This result proved the task was difficult for participants, probably due to the short time for response (similar values were obtained in the previous study using this paradigm, Staudigl and Hanslmayr, 2013). As a dependent variable in the declarative memory task, we used the discrimination index *d* prime (*d*′) calculated from hits and false alarms according to signal detection theory (Macmillan and Creelman, 2005). The numbers of words correctly (hits) and incorrectly (false alarms) recognized as “old” are presented in Table 1, together with the resulting *d* prime value. Independent of sleep or waking, recognition performance was significantly better for words presented in the matching context (0.45 ± 0.05) as compared to words presented in the non-matching context (0.39 ± 0.04, main effect of “context” *F*_(1,42)_ = 5.39, *p* = 0.025, *η*^2^ = 0.114). In contrast to Experiment 1, we did not observe any beneficial effect of sleep vs. wakefulness on recognition performance independent of the context condition (*F*_(1,42)_ = 2.74, *p* = 0.11, *η*^2^ = 0.061), suggesting that recall testing might be more sensitive for the detection of the beneficial effect of sleep as compared to recognition testing. We also did not find any significant interaction between sleep and context even when using recognition testing (*F*_(1,42)_ = 0.09, *p* = 0.77, *η*^2^ = 0.002, see Figure 2B). In each of the word/video pairing combinations (matching, non-matching, new), number of answers given at each confidence level did not differ between the groups (all *p* > 0.16). There were no differences in memory performance between males and females and no interaction of gender with other factors (all *p* ≥ 0.12).

**Table 1:** **Memory task performance in both experiments**.

Group	Condition	Experiment 1	Experiment 2		
		Recalled words M ± SEM	Hits M ± SEM	False alarms M ± SEM	*d* prime M ± SEM
	Matching	5.18 ± 0.39	77.67 ± 2.09	57.46 ± 2.70	0.45 ± 0.05
	Non-matching	4.54 ± 0.38	74.98 ± 2.17		0.39 ± 0.04
Wake	Matching	4.36 ± 0.39	79.35 ± 2.57	54.78 ± 3.21	0.55 ± 0.07
	Non-matching	3.86 ± 0.46	76.09 ± 2.84		0.47 ± 0.06
Sleep	Matching	5.97 ± 0.63	76.00 ± 3.31	60.13 ± 4.34	0.35 ± 0.06
	Non-matching	5.21 ± 0.58	73.87 ± 3.31		0.30 ± 0.06

*Mean numbers of recalled words (Experiment 1), correct and incorrect answers “old” and weighted sensitivity measure d prime (Experiment 2). Maximum score in each context condition was 24 in Experiment 1 and 120 in Experiment 2. M, mean; SEM, standard error of the mean*.

Response-bias measure (*c*) reflecting the degree of response tendency towards answering “old” or “new” was also calculated (see “Materials and Methods” Section). In general, the main response criterion was slightly negative (−0.17 ± 0.05 in matching and −0.14 ± 0.05 in non-matching condition). This affirms a higher tendency to classify a word as old rather than new, possibly reflecting the higher amount of old words than new words in the sample. However, also in this measure we did not find any significant differences between sleep and wake groups and no significant interaction with the context conditions (all *p* > 0.77).

### Control for Possible Circadian Confounds

We did not find any significant differences between morning and evening sessions with respect to subjective indications of mood (good vs. bad), alertness (alert vs. tired) and tension (anxious vs. relaxed) neither in Experiment 1 (all* p* > 0.55), nor in Experiment 2 (all *p* > 0.41). In addition, we did not find any significant differences between morning and evening session in the working memory tasks: Performance in the mathematical task in Experiment 1 was not significantly different between morning and evening session (*p* = 0.34). Subjects’ performance in the N-back (0-back and 2-back) task in Experiment 2 did neither differ in accuracy (all *p* > 0.19), nor in reaction times (all *p* > 0.16). Thus, it is highly unlikely that circadian influences might have confounded our results.

## Discussion

Our results of the two separate experiments consistently show that sleep does not support a decontextualization of memories. As expected, reinstatement of the learning context during recall (Experiment 1) and recognition testing (Experiment 2) improved retrieval performance as compared to the non-matching context conditions. These findings add further evidence to the well-known context effect of memory, robustly producible with movies as context (Smith and Manzano, 2010). Sleep after learning improved recall of words, but not recognition as compared to a retention interval filled with wakefulness, which is consistent with pervious results using either recall or recognition procedures (for an overview, see Diekelmann et al., 2009). Most importantly, we did not find any hint for an interaction between these two main effects of context and sleep on memory, indicating that sleep does not influence the effect size of a reinstated context on memory retrieval. According to our results, sleep after learning does neither weaken nor strengthen the association between the learned item and its learning context. Our results partly contradict the theoretical predictions of an increased semantization and decontextualization of a night of sleep after learning on episodic memories (Lewis and Durrant, 2011). They also contradict results reported by Cairney et al. (2011) which suggested that the influence of context reinstatement during retrieval on memory performance was weakened after a retention interval filled with sleep. However, as outlined in the introduction, the study by Cairney et al. (2011) is confounded by immediate recall testing in the same retrieval context as delayed retrieval conducted before the critical retention period, which might fully explain this discrepant result pattern. Here we decided to omit immediate retrieval testing entirely. However, immediate retrieval allows the examination of the development of context effects on memory over time (see e.g., Cox et al., 2014), which is not possible in our study. Generally, one might argue that sleep-dependent memory effects are due to higher interference to memory occurring during waking rather to sleep *per se*. However, studies designed to include identical amount of waking-associated interference in between the learning and subsequent recall (e.g., Gais et al., 2006) show that beneficial effect of sleep on the memory cannot be explained by this factor. For example, in one of the experiments (Gais et al., 2006, Experiment B) subjects either learned the words in the evening and had a night of sleep or were sleep deprived and went to sleep in the morning, sleeping almost the same amount of time. When tested after one more night and day (48 h after initial learning) they showed better recall when the acquisition was closely followed by sleep rather than wakefulness. Future studies should use a similar design to exclude interference effects on context-dependent memory consolidation and sleep.

Regarding the interaction of context reinstatement and sleep on memory, our results agree with those of Cox et al. (2014) who reported no interaction of context reinstatement and sleep on memory. Furthermore, two studies even reported a strengthening of associations between contextual information and items (Lewis et al., 2011; van der Helm et al., 2011), although these studies did not use a classical context reinstatement procedure but rather a more explicit learning of a “local” item-context association. Also in our studies, the learning context was “local” i.e., the context was different for each of the words. Indeed, it might be possible that local vs. global contextual information are processed differently and have different consequences on later memory retrieval. In particular, local contextual information might be encoded intentionally together with the content, while global contextual information is typically encoded incidentally. However in our study, participants were not aware that the words had to be recalled later and they performed on an unrelated task (i.e., alphabetical judgment). Thus, the incidental learning of contextual information encoding was preserved.

Furthermore, Experiment 1 might bring about some concerns due to the particular form of the recall session. First of all, the unmatched words had to be recalled without a clearly predefined context realized during learning (e.g., reshuffling of learning contexts A and B during retrieval). However, the context effect on memory depends on the availability of contextual cues, matching with those present at encoding. Thus, a new context during retrieval is a condition in which contextual cues are available to a lesser degree. Previous studies show that retrieval performance during a completely new context is comparable to recall during a predefined and reshuffled context (e.g., Herz, 1997; Smith and Manzano, 2010). Therefore, we consider it unlikely that this factor has influenced our results. Second of all, in the Experiment 1 the time for recalling the words was limited by the duration of videos presentation, which might have impeded a full recall for some participants. Even worse, if contextual cue facilitated retrieval of a subset of words, subjects could start from reporting these words and fail to get to the others. However, we would like to emphasize that participants actually recalled a considerable number of words whose videos were not presented at retrieval (Figure 2A). Furthermore, sleep improved recall also for these words and their recall was even (numerically) higher as compared to the number of words recalled in the wake condition whose videos were presented.

Overall, several empirical results including our own now contradict a sleep-dependent decontextualization of memories and the assumed gist extraction process in which associations to sparse additional information is weakened. It must however be considered that most studies measured only one single night of sleep. One sleep period might be insufficient to induce a behaviorally relevant weakening effect on context memory. It might need several nights of consolidation to observe schema formation and context reduction accompanied by a reduced hippocampal involvement. Although it is experimentally challenging to test the effects of multiple nights on contextual memory, some findings indirectly support this assumption: for example, Gais et al. (2007) measured brain activity during cued recall immediately, 2 days and 6 months after a learning task. In a within-subjects design, subjects either slept during the two nights between immediate and second recall or were sleep deprived for 24 h and only slept the second night before recall. When subjects were allowed to sleep after immediate recall, memory performance as well as hippocampal activity was higher 2 days after learning compared to the condition in which they were sleep deprived. In contrast, at retest after 6 months, retrieval preferably recruited the mPFC for words learned before sleep, but not before sleep deprivation. This study hint at an early consolidation stage with a duration of several days or weeks, in which the hippocampus is still critically involved in recall. After several months, the hippocampal contribution then gradually diminishes in favor of a cortically driven activation. Moreover, these results suggest that the redistribution to cortical areas is initiated in the first night of sleep after learning but only detectable after longer time periods (i.e., several months).

In sum, our data supply evidence against a weakening impact of sleep on context effects across one night. Thus, one possible interpretation is that sleep does not actively contribute to the process of decontextualization or semantization as predicted by the active system consolidation theory. On the other hand, one could argue that the first night of sleep after learning solely initiates the process of decontextualization, followed by multiple decontextualization rounds during subsequent nights. Thus, future studies should experimentally manipulate sleep vs. wakefulness during the first 12 h after learning, but then measure retrieval performance after multiple nights. This could be a more sensitive measure of potentially long-lasting processes of decontextualization. Finally, one could also discuss whether context effects on memory are indeed a valid indicator of processes of decontextualization. In fact, context effects on memory are detectable after very long time interval up to 7 years (Aggleton and Waskett, 1999). Thus, it might be theoretically possible that a strong episodic memory traces initially allows successful free recall largely independent of context cues. Thus, the context effect of memory is initially small. With longer retention intervals (i.e several days/weeks), the strength of the “episodic” part of the memory is weakened during the processes of decontextualization, leading to a reduced ability to “freely” recall the information. Here, available contextual cues might facilitate retrieval performance, because the “episodic” nature of the memory becomes weaker. Thus, it might even be possible that stronger context effects of memory are indicative of a *weaker* (instead of stronger) association between content and context of a memory trace. Only at the very end of the decontextualization processes, contextual cues might be ineffective to support (episodic-like) retrieval performance. Thus, future experiments should use both episodic-like and more semantic tasks to more specifically examine the role of sleep in the “decontextualization” of memories.

## Author Contributions

KJ, TS and BR designed the experiments, KJ conducted the experiments, KJ, MJC and BR analyzed the data, all authors wrote the article.

## Conflict of Interest Statement

The authors declare that the research was conducted in the absence of any commercial or financial relationships that could be construed as a potential conflict of interest.
